# Spatial benefit assessment and marine climate response of coastal zone in Fujian Province under cross-system influence

**DOI:** 10.1371/journal.pone.0306988

**Published:** 2024-11-11

**Authors:** Wenjun Chen, Chaoxiang Wen

**Affiliations:** 1 School of Public Affairs, Xiamen University, Xiamen, China; 2 School of Architecture and Civil Engineering, Xiamen University, Xiamen, China; Jinan University, CHINA

## Abstract

To gain a scientific understanding of the cross-system impact of coastal zones and promote the sustainable development and protection of coastal areas, we constructed a spatial benefit evaluation system that encompassed both terrestrial and marine systems, focusing on the ecological, economic, and social dimensions. We employed the entropy method, moving average method, and Mann-Kendall trend test to quantitatively characterize the spatial benefits of the coastal zone in Fujian Province, China, and the evolution of the marine climate from 2005 to 2020. Building on this, the grey relational analysis method was applied to investigate the correlation between spatial benefits and marine climate and to explore the trends and magnitude of the impact of marine climate on spatial benefits. During the study period, the spatial benefits of the coastal zone in Fujian Province exhibited a fluctuating pattern of an initial increase followed by a decrease, with spatial benefits varying among cities. The role of the economic system in enhancing spatial benefits was not considerable. Changes in the marine climate aligned with the global warming trend, with the most considerable changes observed in sea level and tropical cyclone frequency and intensity, which are sensitive to human activities. There was a high degree of correlation between coastal zone spatial benefits and marine climate, with seawater salinity being most closely related to spatial benefits, while tropical cyclones showed the weakest correlation. The results of this study support sustainable development efforts in coastal zones.

## 1. Introduction

Coastal zones are essential interfaces where the land and sea interact, and they play a critical role in maintaining global ecological security and human well-being [1]. Since the 1990s, the rapid development of coastal cities and various human activities have placed considerable pressure on coastal resources. This has resulted in the loss of wetlands, increased pollution, a decline in fishery resources, and reduced biodiversity [2]. Traditional spatial planning practices often fail to consider the interconnected impacts of land and sea, leading to a lack of coordinated planning and use of marine and terrestrial resources, which is not conducive to sustainable ecological development. Therefore, it is crucial to understand the unique cross-system impacts of coastal areas and scientifically evaluate the level of spatial development and utilization to alleviate environmental pressures on coastal space resources and promote the coordinated development of the ecological environment and economy.

In urban planning, early space evaluations were primarily based on economic output and the efficiency of land resources [3]. However, with increasing social and ecological challenges due to urbanization, the focus on land use benefits alone has been questioned as it neglects social and environmental values. This has led to an expanded view of land use benefits, shifting the evaluation system towards a more comprehensive approach and leading to the concept of spatial benefits [4]. Spatial benefits emphasize the comprehensive use and optimized allocation of resources across a broader spatial context, focusing not only on economic gains but also on social equity, environmental quality, and biodiversity. Despite this, the concept of spatial benefits is not well defined, and existing research mainly focuses on large-scale assessments of society, economy, and ecosystems at the national or urban cluster levels [5, 6], with few studies on the spatial benefits of coastal areas [7]. Existing coastal zone evaluations typically only measure the land use benefits of coastal spaces without considering the overall utilization level of spatial resources.

Moreover, as a unique transitional zone between the land and sea, research on the spatial benefits of coastal spaces must account for cross-system interactions. However, existing research has mainly focused on the effects of ocean climate change on the land, the cryosphere, and ecosystems, with limited investigation into how ocean climate affects the spatial benefits of coastal zones through cross-system interactions [8–10] This gap is particularly detrimental to integrated land-sea planning and the sustainable development of coastal spaces. Therefore, there is an urgent need to explore the spatial benefits of coastal zones and their relationship with ocean climate change to inform coastal space planning and design.

The study presents a comprehensive approach to examining the spatial advantages of Fujian Province’s coastal area and its response to marine climate variability. An integrated evaluation system has been developed to capture ecological, economic, and social dimensions, utilizing the entropy method for quantifying spatial benefits. The methodology includes a moving average technique and the Mann-Kendall trend test to track the trajectory of marine climate from 2005 to 2020. Grey relational analysis is then used to measure the correlation between these spatial benefits and marine climate parameters. The anticipated outcomes include a detailed assessment of the coastal area’s spatial advantages, a deeper understanding of the impact of marine climate on them, and evidence-based strategies for sustainable coastal zone management. This research makes novel contributions by scientifically elucidating cross-system impacts on coastal zones and exploring the complex interplay between spatial benefits and marine climate. It advances the field by offering an integrative method for evaluating coastal spatial benefits and their dynamic responses to climate, providing essential insights for policy-making and enhancing coastal resilience.

## 2. Literature review

### 2.1 Benefits of coastal zone spaces

Spatial production theory posits that space serves as a medium for production relations and forces that are shaped by social practices and tailored to specific modes of production [11]. Initially, research viewed space as a "container," highlighting its role in social support and emphasizing its economic aspects [12]. By evaluating the economic value of space, decision-makers can gain a more precise understanding of its financial potential. Spatial benefits, a key measure for assessing the efficiency of coastal resource use, originated from performance concepts in business management that were initially aimed at evaluating quality and outcomes [13]. As research has evolved, the concept of performance has been integrated into fields such as ecology, sociology, and public administration to address various aspects of the environment, the economy, and governance [14, 15]. However, early space assessments focused on the productivity and utility of space, particularly regarding land use and economic yield. As economies grow and regional disparities widen, the constraints of ecological limitations become more pronounced. Theories of postmodern spatial production, spatial justice, and sustainable development have introduced a multifaceted approach to space that considers elements such as power dynamics, cultural influences, and the ecological environment [16]. This has steered spatial evaluation towards a focus on "comprehensive benefits" rather than just "single efficiency," broadening the concept of spatial benefits [17, 18]. These benefits extend beyond the economic value of land use and include optimizing spatial resources, environmental conservation, and social governance across various dimensions.

### 2.2 Cross-system influences in coastal zones

The coastal zone is shaped by a complex interplay of oceanic physical processes, marine resource exploitation, climate change, extreme weather events, and terrestrial production activities [19]. This interaction bestows the coastal zone with unique spatial traits and ecological roles while facilitating the exchange of matter, energy, and information across regional systems [20]. Therefore, for a scientifically rigorous assessment, the evaluation of spatial benefits must incorporate economic, social, and other multifaceted systems and the interplay of land-sea influences [21]. However, current research often employs indicators from natural environments, economic growth, and social demographics, using evaluation models such as the pressure state response framework [22]. These indicators may not be closely related to the unique aspects of coastal zones, and they may not fully capture their distinctiveness. To advance the evaluation of the benefits of coastal zones, it is imperative to integrate the distinctive ecological and environmental features of these spaces, transcending conventional concepts and evaluation limitations. Establishing a systematic evaluation method for coastal zone benefits will enable a more accurate assessment, offer scientific guidance for coastal zone planning, and ensure the realization of sustainable development objectives. This refined approach to spatial benefits in coastal zones is essential for effective decision-making and promotion of balanced ecological and economic progress.

The intricate relationship between the land and sea directly affects the benefits of spatial interactions [23]. When human activities are within natural limits, the equilibrium sustains harmonious system development [24]. However, exceeding these limits leads to competitive dynamics between land and sea development, which disrupts spatial benefits. Thus, evaluating coastal zone benefits should integrate economic, social, and ecological dimensions, rather than simply aggregating individual system benefits [25]. A thorough evaluation requires a meticulous examination of pivotal factors such as marine resource development, ocean climate dynamics, economic growth, and human activities to accurately reflect the use and stewardship requirements of coastal resources. Embracing a cross-system perspective in the assessment captures the distinctiveness of the coastal zone while providing a robust scientific foundation for decision-making in planning and managing these regions.

### 2.3 Oceanic climate regulation

The dynamics of the interactions between terrestrial and marine systems are multifaceted and varied, with effects that differ in nature and scope. These interactions can be broadly classified into terrestrial and marine types. Notably, complex shifts in oceanic climate directly influence the exchange of materials and energy flow across land and sea, exemplifying a quintessential marine influence [26]. In the context of global warming, the disruption of the nutrient balance in coastal waters, the progression of ocean acidification, and the enlargement of oxygen-depleted zones have led to the depletion of fishery resources [27]. Investigating porewater exchange and iron transformation in these systems is increasingly crucial, as they are directly interconnected with the broader ecological and economic well-being of coastal regions [28, 29]. Moreover, the marked increase in sea level has heightened the incidence of saltwater intrusion and the frequency of extreme storm surges, posing substantial challenges to infrastructure development [30].

Conversely, not all manifestations of oceanic climate change are detrimental; they can also result in beneficial outcomes [31]. For example, moderate ocean warming can induce changes in marine phenology, hastening the growth and maturation of marine life, whereas glacial melting can shorten shipping lanes, thereby lowering transportation expenses [32]. Given the complex impacts of oceanic climate change on coastal zones, a comprehensive understanding of the interplay between the oceanic climate and spatial benefits is imperative. It is also critical to develop strategies to adapt to climate change, address cross-system challenges across land and sea, and expand benefits to coastal spaces.

To address the gaps in the existing research, we focused on analyzing coastal zone spatial benefits and marine climate trends, and their underlying connections, to inform and enhance integrated land-sea planning and sustainable development. We investigated a spatial benefit evaluation framework for the coastal zone of Fujian Province, China that quantifies coastal resources and guides future development while aligning ecological preservation with economic growth. Additionally, we examined the complex impacts of marine climate change on the ecological, economic, and social dimensions of coastal zones. Building on this analysis, we explored how the assessment and management of coastal zone benefits can be strengthened through the integration of cross-system land-sea interactions. The results of this study provide a valuable reference for evidence-based policies for the sustainable management of coastal zones and an important resource to assist policymakers, urban planners, and environmental managers in addressing the challenges posed by climate change.

## 3. Materials and methods

### 3.1 Study area

Fujian Province’s coastal zone, a key region of urban agglomeration on the west coast of the Strait of China and the Maritime Silk Road, has increasingly faced issues such as excessive land development and marine environmental degradation due to active human activities. The Fujian coastal zone has an extensive coastline with a total length of 6128 km, including 3324 km of mainland coastline and 2804 km of island coastline, making Fujian the province with the second longest mainland coastline. The region is rich in marine resources and is characterized by frequent human activities. In 2020, the Gross Domestic Product (GDP) of the six coastal cities in Fujian Province reached Chinese Yuan (CNY) 3637.128 billion, accounting for 82.84% of the province’s GDP. From a management perspective, the provincial unit exhibits diverse human activities and a high degree of complexity in the coastal zone. By contrast, county-level units have smaller spatial systems with lower complexity and more discernible mechanisms of cross-system influence, offering greater controllability and operability [33]. Therefore, we focused on the coastal zone of Fujian Province, which includes 35 districts and counties in Ningde, Fuzhou, Putian, Quanzhou, Xiamen, and Zhangzhou.

### 3.2 Evaluation system

Ecological, economic, and social systems form the foundation of the complex coastal zone mega‒system, and evaluating the spatial benefits of coastal zones requires a comprehensive consideration of the collective impact of these multi-dimensional subsystems. The ecological environment serves as a fundamental support for the operation of the coastal zone system and is a prerequisite for development. The economy is a key element in coastal zone development and a direct driving force of its exploitation and utilization. Social progress, the ultimate goal of coastal zone spatial development, is at the core of the system’s operation. Therefore, based on the principles of relevance, feasibility, and operability, 19 relevant indicators were selected from ecological, economic, and social dimensions to construct an evaluation index system for the benefits of coastal zone spaces (see Table 1).

**Table 1:** Evaluation indicators and data sources of coastal zone spatial benefits.

Dimensionality	Index level	Use data and sources
Ecology	Coastal zone carbon sink (kg/a)	National land grid data, photosynthetically active radiation, vegetation effective photosynthetic radiation absorption ratio, normalized differential vegetation index in 2005, 2010, 2015, 2020 (Geographic Data Platform of College of Urban and Environmental Sciences, Peking University, Resource and Environmental Science and Data Center of Institute of Geographic Sciences and Natural Resources Research, Chinese Academy of Sciences, Geographic Remote Sensing Ecological Network Platform, etc.)
	Mariculture area (km^2^)	China Marine Statistical Yearbook (2005‒2020), Environmental Status Bulletin of Fujian Province during 2005‒2020, and Ecological Environment Quality Bulletin of five cities, including Ningde City and Fuzhou City.
	Percentage of sea area of Class Ⅰ and Ⅱ water quality (%)	
	Energy consumption per unit GDP (tee/ (ten thousand yuan)	Fujian Statistical Yearbook (2005‒2020), Statistical Bulletin of National Economic and Social Development of Fujian Province for 2005‒2020, and Statistical Yearbook of urban areas for 2005‒2020.
	Water resources per capita (ten thousand square meters)	
	Sea Water Utilization (ten thousand tees)	Fujian Statistical Yearbook (2005‒2020), Fujian Provincial Environmental Status Bulletin for 2005‒2020, and China Marine Statistical Yearbook (2005‒2020)
	Cumulative Red Tide Area (km^2^)	Fujian Statistical Yearbook (2005‒2020), Fujian Provincial Environmental Status Bulletin for 2005‒2020, and China Marine Statistical Yearbook (2005‒2020)
Economy	Coastal zone GDP (ten thousand yuan)	2005, 2010, 2015, 2020 GDP grid datasets (Geographic Data Platform, School of Urban and Environmental Sciences, Peking University, Resource and Environmental Science and Data Center, Institute of Geographic Sciences and Natural Resources Research, Chinese Academy of Sciences, etc.)
	Proportion of fishery output value to primary output value (%)	China Marine Statistical Yearbook (2005‒2020), Environmental Status Bulletin of Fujian Province during 2005‒2020, and Ecological Environment Quality Bulletin of five cities, including Ningde City and Fuzhou City.
	Revenue of coastal tourism service industry (ten thousand yuan)	
	Regional GDP per capita (Yuan/person)	Fujian Statistical Yearbook (2005‒2020), Statistical Bulletin of National Economic and Social Development of Fujian Province for 2005‒2020, Statistical Yearbook of Urban Areas for 2005‒2020, National Research Network Statistical Database, and China Land and Resources Statistical Yearbook.
	Land tax (ten thousand yuan)	
	Fishery output per unit area (t/km^2^)	Fujian Statistical Yearbook (2005‒2020), Fujian Provincial Environmental Status Bulletin for 2005‒2020, and China Marine Statistical Yearbook (2005‒2020)
	Aquaculture production per unit area (t/km^2^)	Fujian Statistical Yearbook (2005‒2020), Fujian Provincial Environmental Status Bulletin for 2005‒2020, and China Marine Statistical Yearbook (2005‒2020)
Society	Coastal population (persons)	2005, 2010, 2015, 2020 Population grid data sets (Geographic Data Platform, School of Urban and Environmental Sciences, Peking University, Resources and Environmental Science and Data Center, Institute of Geographic Sciences and Natural Resources Research, Chinese Academy of Sciences, etc.
	Per capita sea area (km^2^)	China Marine Statistical Yearbook (2005‒2020), Environmental Status Bulletin of Fujian Province during 2005‒2020, and Ecological Environment Quality Bulletin of five cities, including Ningde City and Fuzhou City.
	The degree of coastline artificialization	
	Natural population growth rate (%)	Fujian Statistical Yearbook (2005‒2020), Statistical Bulletin of National Economic and Social Development of Fujian Province for 2005‒2020, and statistical Yearbook of urban areas for 2005‒2020
	Urbanization level (%)	

The selected data primarily comprised geographical and socioeconomic statistics. Indicators such as the area of marine aquaculture, percentage of sea areas with Class I and II water quality, ratio of fishery output value to the primary output value, and degree of artificialization of coastlines were calculated and obtained through spatial interpolation using ArcGIS 10.4 software. Data on coastal zone carbon sequestration, which relies on land use data, were acquired through operations such as overlay, extraction, and reclassification in ArcGIS to obtain primary and secondary classified land use data for the study period. Furthermore, compared with traditional methods of consulting statistical yearbooks and bulletins, the integration of indicators such as coastal zone carbon sequestration, land tax revenue, and the degree of artificialization of coastlines, which directly reflect the carrying capacity and extent of development and utilization of coastal zone ecological resources, can enhance the scientific validity and credibility of the evaluation results.

### 3.3 Climatic indicators

In alignment with the research timeframe of spatial benefits, we selected six climatic indicators within the maritime scope of Fujian’s coastal zone: sea surface temperature, sea level, tropical cyclone frequency and intensity, sea surface wind speed, seawater salinity, and sea level height (see Table 2). These indicators have short-term physical impacts on nearshore sea areas, extending to the baseline of the territorial sea. Time-series analysis and breakpoint identification were conducted for these indicators. Based on this, we explored the correlation between the oceanic climate and the spatial benefits of Fujian’s coastal zone, thereby identifying the cross-system influences between land and sea. Marine meteorological data, such as sea surface temperature and sea level, were obtained through spatial interpolation based on meteorological station observations, whereas tropical cyclone data were compiled from the China Weather Network, integrating scores based on historical records of cyclone levels and occurrences.

**Table 2:** Summary of marine climate data.

Basic data	Data source
Sea water temperature (°C)	The 30 m annual land cover datasets and its dynamics in China from 1990 to 2021 (Zenodo Open Scientific Data Repository)
Salinity of seawater (PSU)	From 2005 to 2020 global Marine Argo grid data set (https://argo.ucsd.edu/data/argo‒data‒products/)
Sea level(hPa)	Daily mean sea level data from weather stations worldwide, 1929‒2022 (National Center for Environmental Information (NCEI), National Oceanic and Atmospheric Administration)
Sea surface wind speed (m/s)	Daily wind speed Data of China from 2000 to 2020 (Daily Value Dataset of Surface Climatological Data of China)
Tropical cyclone	China Meteorological Network (https://e.weather.com.cn).
Relative height of sea level(m)	From 2005‒2020 global Marine Argo grid data set (https://argo.ucsd.edu/data/argo‒data‒products/)

### 3.4 Methods

#### 3.4.1 Evaluation logic framework

**Fig 1:**
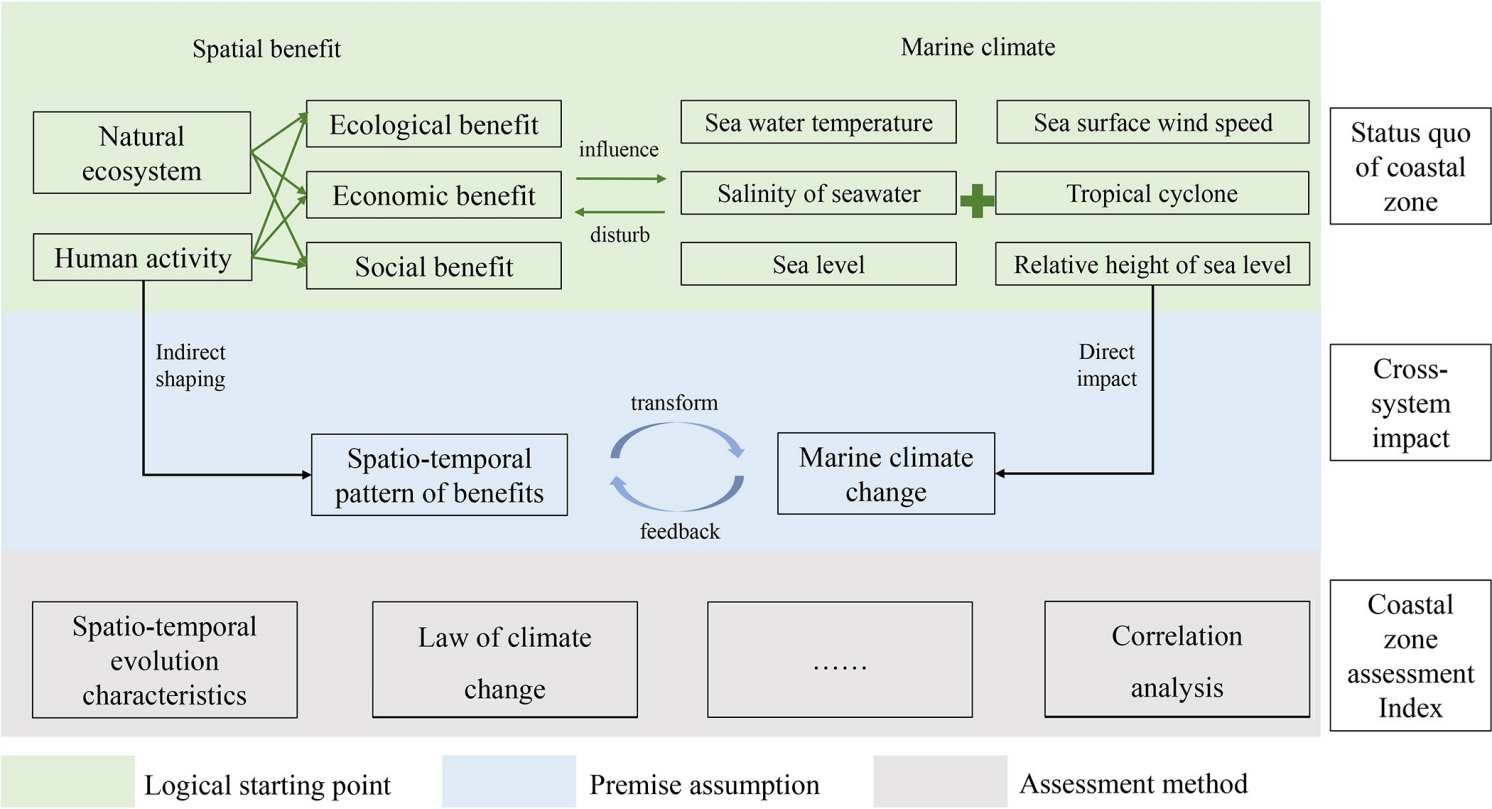
Coastal zone space benefits and a systematic framework for assessing marine climate.

The study examines the coastal zone of Fujian Province, emphasizing the necessity of considering marine climate change in coastal planning and management. It analyzes the spatial benefits at the land-sea interface and their interaction with marine climate change (see Fig 1).

1. Logical Starting Point: Located at the interface of terrestrial and marine environments, the coastal zone of Fujian Province is a critical area where spatial advantages are shaped by a multitude of factors, including natural conditions, ecosystem health, and the significant impact of human activities on marine climate change. The spatial benefits reflect its diverse value in terms of ecological integrity, economic prosperity, and social well-being; they play a pivotal role in evaluating the region’s sustainable development trajectory.
2. Premise assumption: The study proposes a close relationship between the spatial benefits of the coastal zone and marine climate change. These benefits are viewed as a comprehensive representation of the area’s ecological richness, economic strength, and societal well-being, all of which are intricately linked to the fluctuations in marine climate. Elements of the marine climate, such as sea water temperature, sea level pressure, and the frequency and intensity of tropical cyclones, are believed to significantly impact the spatial benefits of the coastal zone by altering its natural environment and human activities. Furthermore, it is suggested that the coastal zone’s spatial structure and development paths also have reciprocal effects on local nuances in marine climate, forming a complex system of mutual influence.
3. Assessment Method: Based on these assumptions and supported by a logical starting point, a robust assessment framework has been carefully designed to encompass ecological, economic, and social dimensions comprehensively. Using various analytical methods including the entropy method, moving average method, and Mann-Kendall trend test, the study quantifies Fujian’s coastal zone’s spatial benefits from 2005 to 2020 while analyzing evolutionary trends and key shifts in a marine climate. The grey relational analysis method is utilized to assess how interconnected these two critical spheres are, revealing detailed interdependencies and impacts between them. The combination of these methodologies reveals intricate interactions between the spatial benefits of the coastal zone and the broader marine climate change canvas.

#### 3.4.2 Entropy method

The entropy method is an objective weighting method that determines the weight value of different indicators based on their correlation with the original data of each indicator. This approach can objectively reflect the weight of each indicator and effectively avoid the bias caused by subjective factors [34]. To make the weights of selected indicators more scientifically and rationally determined, the entropy method is adopted to determine the weights of indicators based on dimensionless. The calculation formula is as follows:

$$e_{j}=-k\sum_{i=1}^{m}y_{ij}lny_{ij},\;k=1/lnm$$ (1)

$$d_{j}=1-e_{j}$$ (2)

$$W_{j}=d_{j}/\sum_{i=1}^{m}d_{j}$$ (3)

$$U=\sum_{i=1}^{m}y_{ij}W_{j}$$ (4)

where: *e*_*j*_ is information entropy; *m* is the number of research units; *k* is the reciprocal of ln (*m*); *y*_*ij*_ stands for the composite score of the *i‒th* system and the *J‒th* index; *d*_*j*_ is the information utility value; *W*_*j*_ is the index weight; *U* is the overall score.

#### 3.4.3 Moving average method

The moving average method is to calculate the moving average in a long series of year-by-year data by sequential increase and decrease. This method is usually used to remove the outliers in the original data and avoid the interference caused by the outliers in the original data to the research results [35]. The calculation formula is as follows:

$${\bar{X}}_{j}=\frac{1}{k}\sum_{i=1}^{n}X_{i+j=1},(j=1,2,\ldots n-k+1)$$ (5)

where: *k* is the sliding length, and 5 is taken in this paper.

#### 3.4.4 Mann-Kendall trend test

Mann-Kendall method is a non-parametric statistical test method, which can not only find out the mutation points in the sequence but also test the changing trend of the sequence [36]. For a time series *X* with *n* sample sizes, construct an order column:

$$S_{k}=\sum_{i=1}^{k}r_{i}$$ (6)

$$r_{i}=\left\{ \begin{array}{l} 1\;x_{i}>x_{j}\\ 0\;else \end{array}\right.\;(j=1,2\ldots ,i)$$ (7)

where: *S*_*k*_ represents the cumulative number of the *i* th sample *x*_*i*_>*x*_*j*_.

Under the assumption of random independence of the time series, the statistics are defined:

$${UF}_{k}=\frac{\left[S_{k}-E(S_{k})\right]}{\sqrt{Var(S_{k})}}\;(k=1,2\ldots ,n)$$ (8)

where: *UF*_1_ = 0, *E*(*S*_*k*_) and *Var*(*S*_*k*_) are the mean and variance of the cumulative count (*S*_*k*_) respectively, and their algorithms are as follows:

$$E(S_{k})=\frac{n(n-1)}{4}\;(k=1,2\ldots ,n)$$ (9)

$$Var(S_{k})=\frac{n(n-1)(2n+5)}{72}\;(k=1,2\ldots ,n)$$ (10)

or a given significance level *α*, e normal distribution table, if |*UF*_*k*_|>*UF*_*α*_,indicates that the trend of the series is obvious. In time series *X* reverse order *X*_*n*_, *X*_*n‒1*_, *…*, *X*_*1*_, then repeat the above process, and at the same time make *UB*_*k*_ *= ‒UF*_*k*,_ (= n, n– 1 …,1), *UB*_1_ = 0.

When *UF*_*k*_ exceeds the critical line, it indicates that the time series has a significant upward or downward trend. If two curves intersect, and the intersection is between the critical boundary, then the time corresponding to the intersection is the time when the mutation begins. Given the significance level α = 0.05, the critical value *U*_0.05_ = ±1.96.

#### 3.4.5 Grey relational analysis

Grey relational analysis is a method to measure the degree of correlation between factors according to the same or different development trends of historical data series between factors [37]. In this paper, the gray correlation degree is used to characterize the similarity and evolution trend between the coastal zone spatial benefits and Marine climate systems. The calculation formula is as follows:

$$\bar{S}=\frac{1}{n}\times \sum_{t=1}^{n}S(t)$$ (11)

where: *Si(t)* is the correlation coefficient of comparing the Marine climate factors of the series to the spatial benefits of the reference series in the year *t*, and the calculation formula is as follows:

$$S(t)=\frac{\Delta_{min}+\rho \times \Delta_{max}}{\Delta (t)+\rho \times \Delta_{max}}$$ (12)

where: *Δ*_*min*_ and *Δ*_*max*_ are the minimum and maximum absolute difference between the comparison and the reference sequence in year *t*, respectively; *Δ(t)* is the absolute difference between the comparison and the reference sequence in year *t*; *ρ* is the resolution coefficient, generally between 0 and 1, usually 0.5.

## 4. Results

### 4.1 Evaluation of spatial benefits

**Fig 2:**
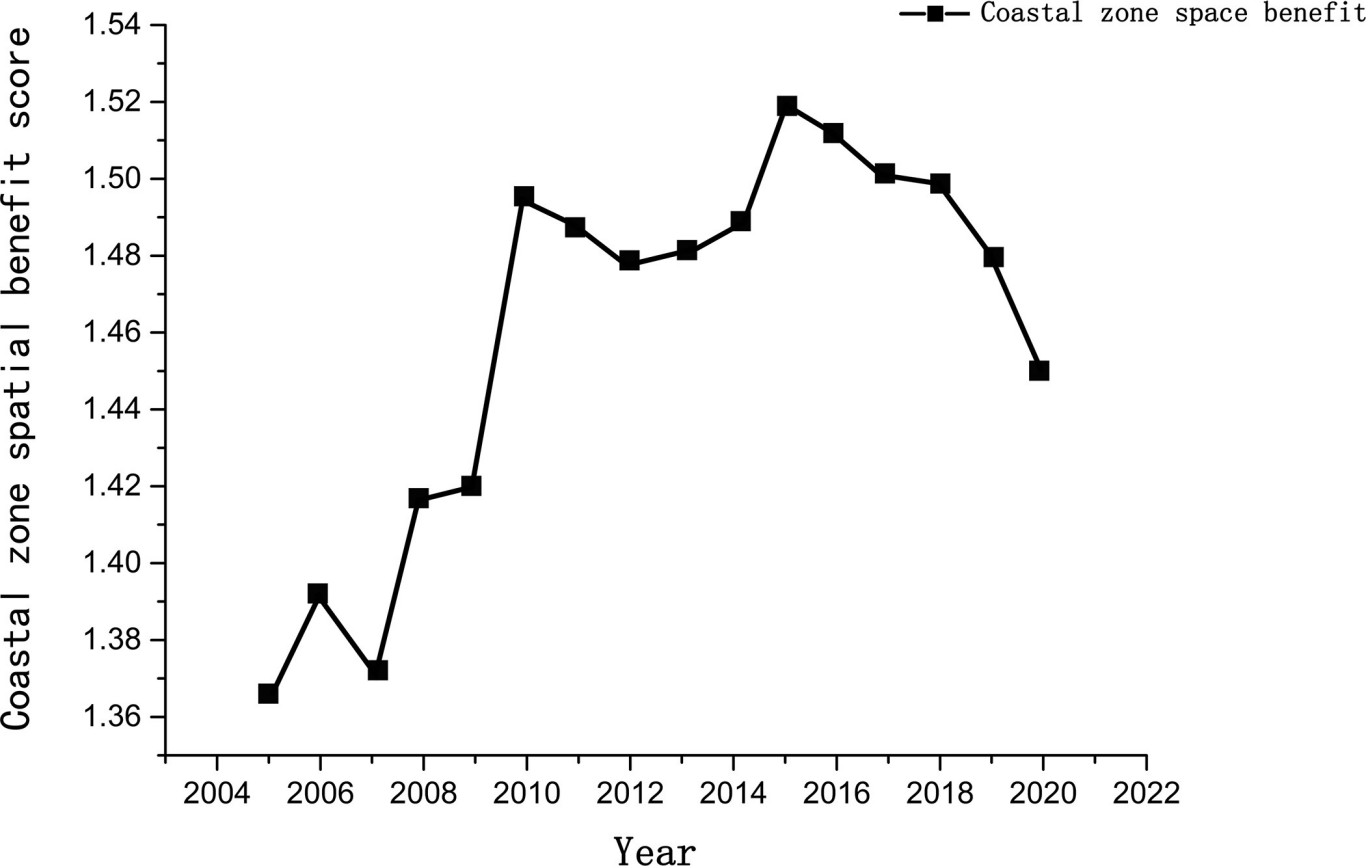
Spatial efficiency scores of coastal zones in six coastal cities of Fujian Province from 2005 to 2020.

The spatial benefits in Fujian’s coastal zone from 2005 to 2020 showed an overall trend of an initial increase, followed by a decline (see Fig 2). The upward trend was most pronounced between 2005 and 2015, peaking in 2015. During this period, ocean development became increasingly active and land reclamation continued to expand. Between 2005 and 2015, the land reclamation area in Fujian reached 2 × 10^4^ km^2^, ranking first in the nation [38]. Large-scale reclamation projects have facilitated economic and social development and considerably enhanced the spatial benefits of coastal zones. From 2015 to 2020, with growing attention to marine environmental protection, policies such as the "Coastline Protection and Management Measures," "Land Reclamation Control Measures," and "Fujian Province Marine Environmental Protection Plan (2011–2020)" were introduced. During this time, sustainable development became a key strategic direction for ocean use, and awareness of ecological protection was further strengthened. However, the marine economic structure and resource utilization methods were not rapidly optimized, and economic and social development faced certain limitations, leading to a decrease in spatial benefits.

**Fig 3:**
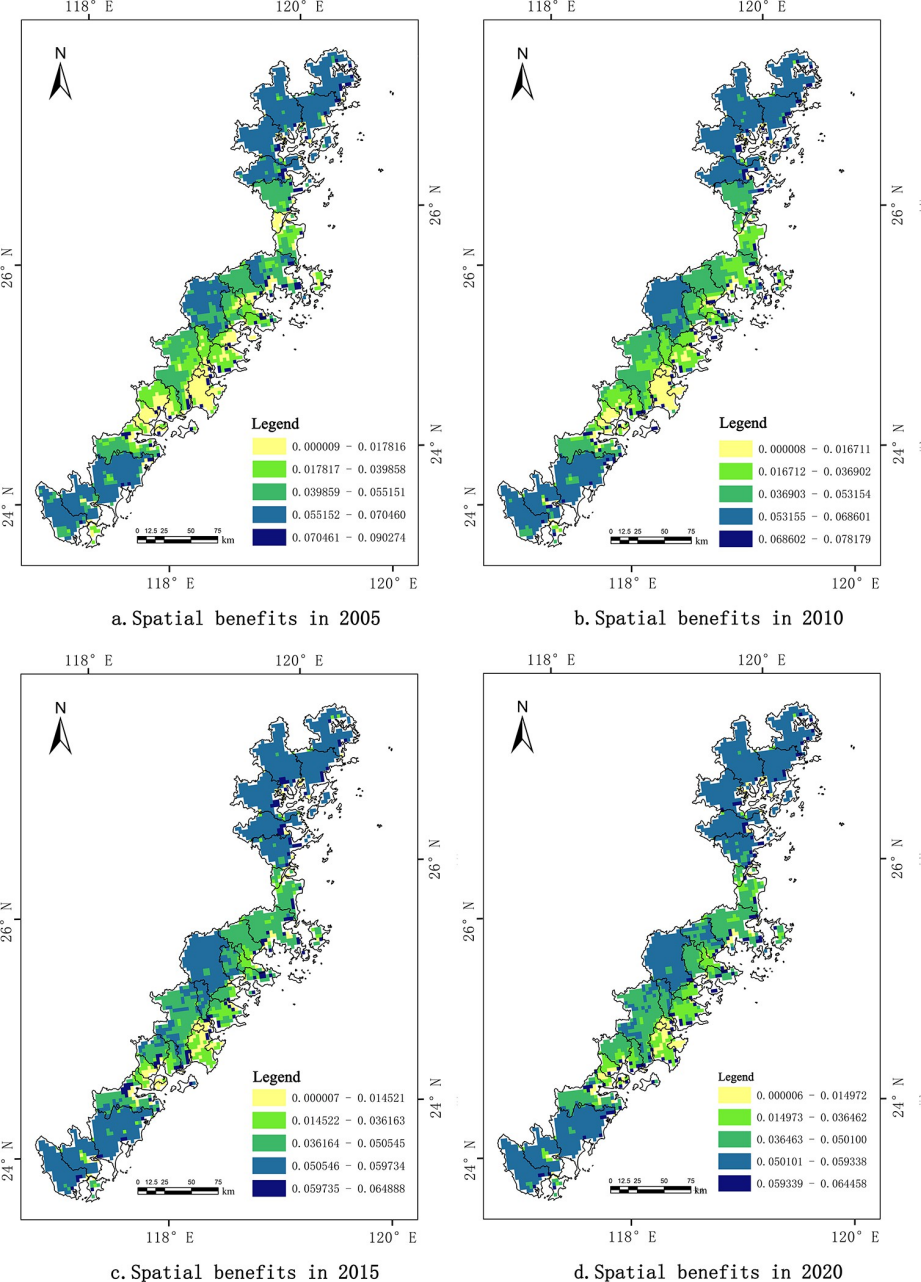
Evolution of coastal zone spatial pattern from 2005 to 2020. (a. Coastal zone spatial benefits in 2005, b. Coastal zone spatial benefits in 2010, c. Coastal zone spatial benefits in 2015, d. Coastal zone spatial benefits in 2020) Note: All administrative boundary data comes from Resource and Environment Science and Data Center https://www.resdc.cn/.

From the evolution of the spatial pattern (see Fig 3), the spatial benefits of the coastal zone in Fujian Province overall presented a distribution pattern described as "three highs between two lows," with Fuzhou, Quanzhou, and Xiamen having relatively lower spatial benefits than the surrounding cities. In 2020, the GDP of these three cities accounted for 60.50% of the Fujian Province’s total, and Quanzhou and Fuzhou individually surpassed the trillion yuan threshold in economic terms. They also hosted 53.53% of the province’s permanent population, reflecting rapid socioeconomic development. However, the calculated spatial benefits of their coastal zones were substantially lower than those of Ningde, Putian, and Zhangzhou, which deviated from general expectations. Between 2005 and 2010, Fuzhou, Xiamen, and Quanzhou were ranked first, third, and fourth, respectively, in terms of land reclamation areas [39]. Substantial land reclamation projects have caused severe ecological damage to coastal wetlands and nearshore marine environments, leading to problems such as wetland encroachment, imbalances in land reclamation and compensation, and a decline in seawater environmental quality. These issues have seriously limited the benefits to economic and social systems. Moreover, influenced by the rapid urbanization process, the contradiction of having a large population with limited land has become increasingly prominent in these three cities, lowering the overall spatial benefits.

### 4.2 Evolution of oceanic climate

**Fig 4:**
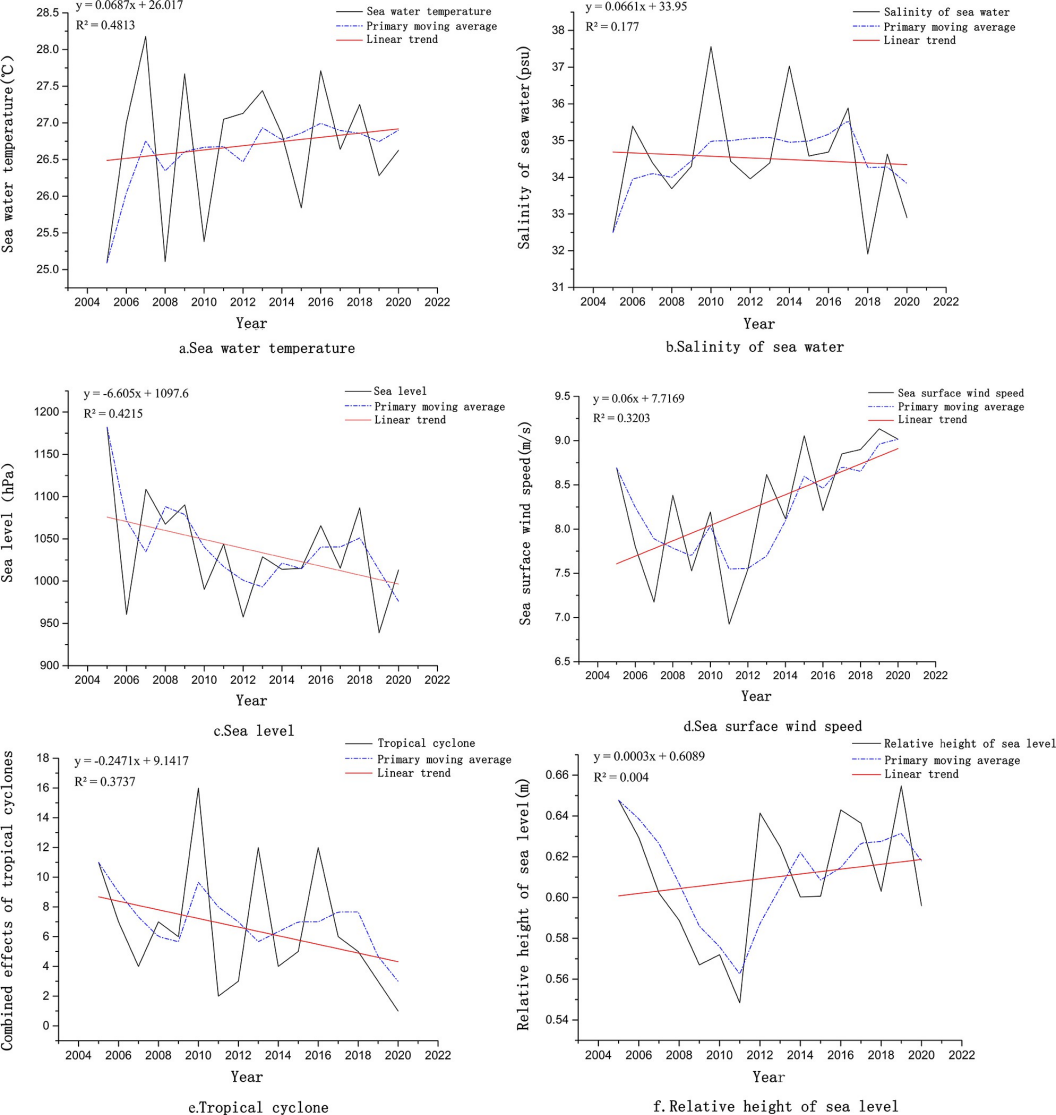
Marine climate change trends in Fujian Province from 2005 to 2020. **(**a. Sea water temperature trend, b. The salinity of seawater trend, c. sea level trend, d. Sea surface wind speed trend, e.Combined effects of tropical cyclones trend, f. Relative height of sea level trend).

The trends in the oceanic climate within the maritime scope of Fujian’s coastal zone from 2005 to 2020 showed inconsistencies. Seawater temperature (see Fig 4A), sea surface wind speed (see Fig 4D), and relative sea level height (see Fig 4F) exhibit a clear upward trend, while seawater salinity (see Fig 4B), sea level (see Fig 4C), and the frequency and intensity of tropical cyclones (see Fig 4E) show a significant downward trend. However, these trends were consistent with the general pattern of oceanic climate change in the context of global warming. Specifically, the increase in seawater temperature and water vapor content led to more frequent and intense precipitation, and the accelerated melting of glaciers contributed to a rise in relative sea level height and a decrease in seawater salinity. Additionally, influenced by global warming, the temperature difference between warm and cold air currents has increased, resulting in higher sea surface wind speeds. Generally, rising temperatures lead to the expansion of the atmosphere, increasing atmospheric mass and causing sea level to rise, forming low-pressure areas that promote the formation and intensification of tropical cyclones. However, in Fujian Province, set against the broader context of climate warming, there was a noticeable trend of decreasing sea level in nearshore areas, along with a reduction in the frequency and intensity of tropical cyclones. Concurrently, variations in wind velocities, oceanic currents, and atmospheric circulation also influenced the trajectories of tropical cyclones, albeit to varying degrees. Changes in wind speed, ocean currents, and atmospheric circulation patterns also altered the paths of tropical cyclones to some extent.

**Fig 5:**
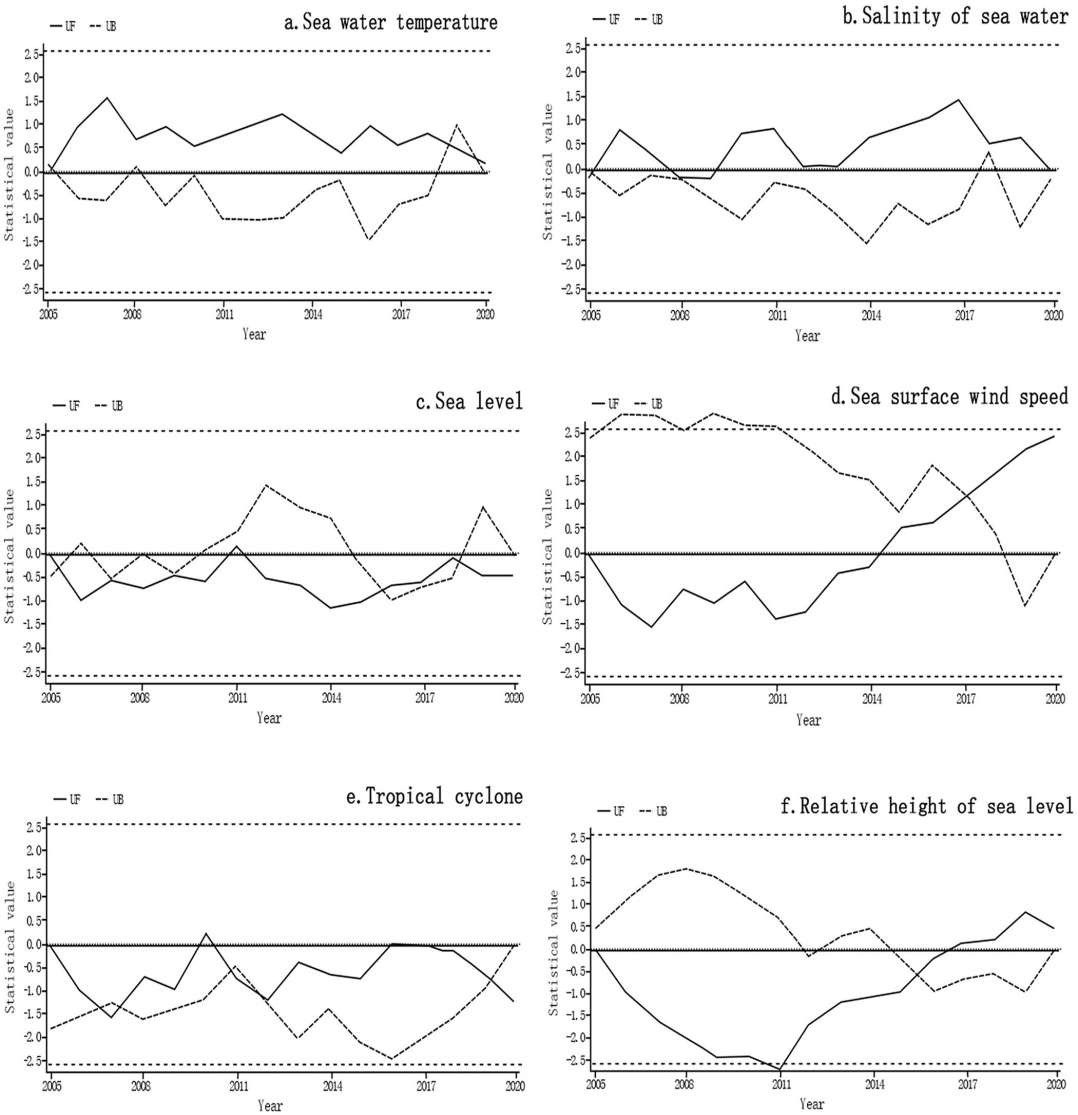
Abrupt climate change in the coastal zone of Fujian Province from 2005 to 2020. **(**a. Sea water temperature mutation analysis, b. The salinity of seawater mutation analysis, c. sea level mutation analysis, d. Sea surface wind speed mutation analysis, e. Tropical cyclone mutation analysis, f. Relative height of sea level mutation analysis).

To further investigate the characteristics of marine climate change in Fujian’s coastal zone, the Mann-Kendall trend test was used for abrupt change detection (see Fig 5). The findings revealed that there were marked abrupt changes in Fujian’s marine climate, with notable changes occurring in 2005, 2007, 2008, 2009, 2011, 2012, 2016, 2017, 2018, and 2019. Notably, sea level and tropical cyclones showed the most frequent abrupt changes (see Fig 5C and 5E), indicating that these factors were sensitive to human activities. This provides a further explanation for the discrepancies in the variations of these factors from the general trends. Moreover, there were single abrupt changes in the sea surface wind speed and relative sea level height (see Fig 5D and 5F), with relatively stable changes observed during the study period. Sea surface temperature and seawater salinity both exhibited two abrupt changes (see Fig 5A and 5B), with the change points occurring during 2005–2010. This suggests that during the early rapid phase of urbanization, changes in underlying surface features such as roads, buildings, soil, and water bodies in the coastal zone had a considerable impact on the marine climate.

### 4.3 Oceanic climate response

The grey relational analysis model was used to analyze the oceanic climate factors that may influence changes in the benefits of coastal zone spaces (see Table 3). The grey absolute relational degrees ranged from 0.3532 to 0.7725, with varying levels of association, except for that of tropical cyclones, which showed a higher degree of correlation. However, the results may be affected when there is a large difference in absolute relational degrees. Therefore, further analysis of the grey relative relational degrees between the two systems was conducted.

**Table 3:** Grey correlation degree between marine climate and spatial benefit.

Marine climatic factor	Absolute correlation degree	Relative correlation degree	Composite value
Salinity of seawater	0.7725	0.8156	0.7941
Sea level pressure	0.7304	0.7885	0.7595
Sea surface wind speed	0.7221	0.7713	0.7467
Sea water temperature	0.7213	0.7438	0.7326
Relative height of sea level	0.7146	0.7289	0.7218
Tropical cyclone	0.3532	0.3436	0.3484

The results indicated that the grey relative relational index between oceanic climate and spatial benefits ranged from 0.3436 to 0.8156, which is close to the absolute relational degrees, suggesting a high level of credibility in the findings. Seawater salinity was the factor most closely related to the spatial benefits of Fujian’s coastal zones. Seawater salinity in Fujian’s coastal zone has shown a decreasing trend in recent years. Lowered seawater salinity can lead to saltwater intrusion inland, posing a threat to the freshwater supply of coastal communities and increasing the risk of over-extraction of groundwater and land subsidence, which would directly impact social development and infrastructure. Additionally, reduced seawater salinity can alter the nutrient structure of nearshore waters, affecting the soil quality in farmlands, mangrove ecosystems, agriculture, and fisheries, thus directly influencing the ecological and economic dimensions of spatial benefits. The frequency and intensity of tropical cyclones were least closely related to their spatial benefits. This is because, despite their seasonal, uncertain, and destructive nature, the effects of tropical cyclones can still be mitigated through dynamic monitoring, scenario simulation, and the establishment of periodic plans; hence their lower degree of association with spatial benefits.

## 5. Discussion

The development and evolution of coastal zone spaces are intricate processes characterized by substantial uncertainty and complexity [40], with spatial benefits influenced by a combination of ecological, economic, and social factors [41]. Traditional perspectives often emphasize the predominant impact of economic factors on spatial benefits [42, 43]. Scholars such as Hu Q P et al (2023) and Wang K et al (2020) have empirically confirmed that economic factors play the most crucial role in and have the greatest influence on the urbanization process of Fujian Province [44, 45]. However, our comprehensive analysis of Fujian’s coastal zone revealed a counterintuitive finding: Large-scale development and construction activities did not effectively enhance spatial benefits. Instead, ecosystem health exerted a decisive constraint on the growth of spatial benefits. This discovery challenges the conventional priority given to economic development and offers a new perspective on the management of coastal zone spaces. For the development of Fujian’s coastal zone, striking a balance between development and ecological protection is key to stimulating new momentum for sustainable economic growth and promoting a shift toward higher-quality economic development. Such a strategic shift will not only enhance spatial benefits but also provide a viable path for achieving both economic development and environmental protection.

With mounting evidence of the impacts of global warming, scholars have recently shifted their focus to examining the effects of climate change on coastal zones [46]. However, current research predominantly emphasizes threats to coastal zone ecosystems and socioeconomic systems from marine disaster events, such as rising sea temperatures, rising sea levels, tropical cyclones, and storm surges [47, 48]. Seawater salinity is a key factor in the coastal zone of Fujian. Nevertheless, the existing integrated coastal zone management primarily prioritizes comprehensive disaster prevention measures. Considering the strong correlation between seawater salinity and spatial benefits, planning and management of Fujian’s coastal zone should prioritize water resource sustainability. This can be achieved by protecting water source areas and constructing reservoirs and water storage facilities to reduce urban reliance on single water sources [49]. In addition, establishing monitoring and early warning systems to detect changes in water quality and salinity is essential, along with implementing strict water resource management policies and regulations. Adopting comprehensive long-term strategies can effectively mitigate the risks associated with decreasing seawater salinity [50]. Our findings also highlight that sea level and tropical cyclone stability are particularly vulnerable areas that require adequate attention during construction phases. The concepts of adaptation threshold and path proposed by the Dutch Climate Change Adaptive Water Resources Management Plan can be used as a reference to formulate the spatial planning of the Fujian coastal zone to support climate change adaptation. The results of this study not only provide new insights for sustainable development but also serve as a valuable reference for global coastal zone management.

It is important to recognize that because of the availability of data and variations in socioeconomic statistical approaches, the selected data indicators can only broadly reflect the trend of changes in the spatial benefits of Fujian’s coastal zone, which may deviate from the actual situation. Furthermore, the impact of oceanic climate events on spatial benefits is not directly linear. This study empirically tested the nearshore climate factors that have a direct effect on the coastal zone, while the indirect impacts of global climate changes, such as atmospheric circulation, El Niño Southern Oscillation, and La Niña events, on spatial benefits were not deeply explored. Future research should consider a wider range of environmental and socioeconomic factors as well as finer spatial resolutions and longer time series. Additionally, owing to the complexity and diversity of land-sea cross-system influences, we only analyzed the association between oceanic climate and spatial benefits. Future studies should continue to explore different types of influencing factors. Nevertheless, this research is a valuable attempt to understand the theory of land-sea cross-system influences. These results elucidate the evolution of spatial benefits in Fujian’s coastal zone and their relationship with the oceanic climate. They provide a scientific basis for the sustainable development of coastal zones, formulation of ecological protection policies, and implementation of adaptation strategies for climate change.

## 6. Conclusions

In this study, we developed a spatial benefit evaluation system that incorporated ecological, economic, and social dimensions and used it to assess the spatial benefits of Fujian’s coastal zone from 2005 to 2020 using the entropy method. The research methods primarily included the entropy method, moving average technique, Mann-Kendall trend test, and grey relational model. The entropy method was utilized to quantify the spatial benefits of the coastal zone, while the moving average technique and Mann-Kendall trend test were employed to analyze the trends in oceanic climate evolution. The grey relational model was then applied to evaluate the correlation between the spatial benefits and oceanic climate. We aimed to address the gap in the existing literature regarding the evaluation of spatial benefits in coastal zones and their relationship with oceanic climate change. The main conclusions are as follows:

1. The trend in spatial benefits in Fujian’s coastal zone can be divided into two phases: an ascending phase from 2005 to 2015 and a descending phase from 2015 to 2020. During the first phase, active development and construction in the coastal zone, driven by land use growth and population aggregation, led to substantial regional development and enhanced spatial benefits. In the second phase, with increased ecological awareness and policy regulations, the pace of socioeconomic development slowed down, resulting in a decline in spatial benefits. Spatially, the benefits exhibited a "The three high-value zones are interspersed with two low-value zone" distribution, with Fuzhou, Quanzhou, and Xiamen becoming "basins" of spatial benefits due to large-scale development, human–land conflicts, and severe ecological degradation.
2. The evolution of the oceanic climate in Fujian’s coastal zone was largely consistent with global warming trends. Seawater temperature, sea surface wind speed, and sea level height showed increasing trends, whereas seawater salinity, sea level, and tropical cyclone frequency and intensity decreased markedly. Among these, sea level and tropical cyclones experienced the most frequent abrupt changes and were highly sensitive to human activity.
3. There was a high correlation between coastal spatial benefits and oceanic climate, with a correlation index ranging from 0.3436 to 0.8156, except for the factor of tropical cyclones, which can be mitigated through various preventive and intervention measures during planning and construction. Thus, by reducing their association with spatial benefits, the correlation of other meteorological factors with spatial benefits exceeded 0.7212, indicating a strong impact on the ecological, economic, and social systems of the coastal zone.

This study has made significant progress in evaluating the spatial benefits of Fujian’s coastal zone and its response to marine climate variability. However, it is important to acknowledge the inherent limitations. While the selected indicators are comprehensive, they may not fully capture the complexity of coastal systems, and the methods used are constrained by available data and temporal scales. Furthermore, deeper investigation is needed into the indirect impacts of global climate phenomena on spatial benefits. In conclusion, there is a recognition of the need for future research to expand the scope of environmental and socioeconomic factors, incorporate finer spatial resolutions, and extend time series for a more robust analysis. It is also suggested that advancing the understanding of land-sea cross-system dynamics through higher-resolution satellite data, machine learning techniques, and interdisciplinary collaborations will pave the way for more nuanced coastal zone management strategies. This will enhance predictive capabilities and contribute to the global discourse on sustainable coastal development and climate change.

## Supporting information

S1 Data (XLSX)

S2 Data (XLSX)
